# Comparing Self-Administered Outpatient Parenteral Antimicrobial Therapy (S-OPAT) with Alternative Delivery Models: Risk Factors for Treatment Failure and Complications in a UK Cohort

**DOI:** 10.3390/pathogens15090981

**Published:** 2026-09-15

**Authors:** Oyewole Christopher Durojaiye, Charlotte Fiori, Kamille Jaff Maulion, Evangelos I. Kritsotakis

**Affiliations:** 1 Department of Infection and Tropical Medicine, Sheffield Teaching Hospitals NHS Foundation Trust, Sheffield S10 2JF, UK; 2 Department of Microbiology, University Hospitals of Derby and Burton NHS Foundation Trust, Derby DE22 3NE, UK; 3 Outpatient Parenteral Antimicrobial Therapy (OPAT) Unit, University Hospitals of Derby and Burton NHS Foundation Trust, Derby DE22 3NE, UK; charlotte.fiori@nhs.net (C.F.); kamille.maulion@nhs.net (K.J.M.); 4 Laboratory of Biostatistics, School of Medicine, University of Crete, 71003 Heraklion, Greece; e.kritsotakis@uoc.gr

**Keywords:** complications, outpatient parenteral antimicrobial therapy, S-OPAT, self-administration, treatment outcome

## Abstract

Self-administered outpatient parenteral antimicrobial therapy (S-OPAT) is an increasingly recognised antimicrobial delivery model, yet comparative evidence on its safety and effectiveness remains limited. We compared clinical outcomes across OPAT delivery models and examined predictors of adverse outcomes in S-OPAT. Adult OPAT episodes at a large UK teaching hospital (January 2023–January 2026) were categorised as S-OPAT, healthcare professional home-administered (H-OPAT), or clinic-based (C-OPAT). Primary outcomes were treatment failure, complications, and 30-day unplanned hospitalisation. Outcome risks were compared using robust multivariable Poisson regression with generalised estimating equations and Bonferroni correction. Among 1064 OPAT episodes, 286 (26.9%) were S-OPAT, and cure/clinical improvement occurred in 912/1064 (85.7%). Treatment failure occurred in 26/286 (9.1%) S-OPAT episodes, with no significantly higher risk than H-OPAT (adjusted relative risk [aRR], 0.72; 98.3% CI, 0.43–1.19) or C-OPAT (aRR, 0.94; CI 0.48–1.84). Rates of unplanned hospitalisation and OPAT-related complications were broadly comparable, except potentially vascular access-related events. Within S-OPAT, older age, higher comorbidity burden, diabetes, peripheral vascular disease, and central venous access were associated with treatment failure. Overall, these findings suggest that S-OPAT can be delivered safely and effectively to appropriately selected patients, while enhanced monitoring may be warranted for older individuals and those with greater comorbidity burden or central venous access devices.

## 1. Introduction

Outpatient Parenteral Antimicrobial Therapy (OPAT) has evolved substantially over the past decade and is now an established model of care that enables clinically stable patients requiring prolonged or complex courses of intravenous (IV) antimicrobial therapy to be managed outside the inpatient setting. OPAT can facilitate earlier hospital discharge, reduce hospital bed utilisation, and improve patient convenience, while potentially reducing healthcare costs and other resource requirements [1,2,3].

Traditionally, OPAT has been delivered through several models, including administration of therapy by a healthcare professional—such as a community nurse—in the patient’s home or a community setting, and clinic-based OPAT, where patients attend a healthcare facility (e.g., an ambulatory care unit or infusion clinic) for antimicrobial administration.

In recent years, self-administered OPAT (S-OPAT)—in which patients (or their caregivers) administer parenteral antimicrobials independently at home following appropriate training—has gained increasing attention. S-OPAT provides greater patient autonomy and flexibility [4], facilitates the use of antimicrobials requiring multiple daily dosing, and has a significantly lower environmental footprint than other OPAT models [5]. Despite these advantages, S-OPAT remains underutilised, potentially due to concerns about its clinical outcomes, treatment adherence, and safety compared with other OPAT delivery models [6,7].

Although previous studies have generally suggested that appropriately selected patients can safely undertake S-OPAT, the evidence remains heterogeneous, particularly regarding the factors associated with treatment failure, complications, and unplanned hospitalisation [7]. Comparative studies evaluating S-OPAT alongside both healthcare professional-administered home OPAT and clinic-based OPAT within the same service are relatively limited. A clearer understanding of the relative effectiveness and safety of these delivery models, together with the identification of patients at increased risk of poor outcomes during S-OPAT, may help optimise OPAT delivery and support the development of more individualised and risk-stratified approaches to patient selection [8].

In this study, we therefore evaluated the safety profile and clinical outcomes of S-OPAT compared with other OPAT delivery models within a large tertiary referral teaching hospital in Derbyshire, UK. We also examined patient- and treatment-related factors associated with treatment failure, OPAT-related adverse events, and 30-day unplanned hospitalisation among patients who received S-OPAT.

## 2. Materials and Methods

### 2.1. Study Design

We conducted a retrospective cohort study of all adult patients who received OPAT at University Hospitals of Derby and Burton (UHDB) (England, UK) between January 2023 and January 2026. UHDB is one of the largest NHS trusts in the UK, comprising approximately 1700 inpatient beds and serving a population of more than one million across South Derbyshire and South-East Staffordshire.

The study size was determined by the number of eligible patients during the study period without a formal statistical sample size calculation. Clinical outcomes were assessed at the completion of IV antimicrobial therapy. The study was approved by the local clinical audit and effectiveness team as part of ongoing clinical governance, service development, and evaluation.

### 2.2. Setting

The OPAT service, established in 2013, has been described previously [9,10]. It is delivered by a multidisciplinary team comprising infection specialists, antimicrobial pharmacists, clinical nurse specialists, and community nurses. The service maintains electronic databases to prospectively record patient demographics, treatment regimens, indications and duration of therapy, antimicrobial delivery model, clinical outcomes, and any associated complications. Antimicrobial therapy was provided through three distinct pathways: administration in the patient’s home by community nurses (H-OPAT); daily attendance at a dedicated OPAT clinic (C-OPAT); and self- or carer-administration at home following appropriate training (S-OPAT). A wide range of antimicrobials was administered, including continuous infusion via pre-filled disposable elastomeric pumps for flucloxacillin and piperacillin/tazobactam [9,11].

Patients were referred to OPAT from admission units, inpatient wards, and outpatient clinics. Additional cases were identified through daily review of inpatient antimicrobial prescriptions and discussion at multidisciplinary infection meetings. All patients with infections requiring prolonged IV antimicrobial therapy were considered for OPAT, provided they had stable clinical parameters, no requirement for continuous inpatient monitoring (e.g., uncontrolled sepsis), and no outstanding surgical needs. Patient selection and formulation of individualised OPAT treatment plans were undertaken by the OPAT infection specialists. The choice of delivery model was guided by clinical, logistical, and patient-centred considerations, including clinical stability, functional ability, home circumstances, proximity to the OPAT clinic, capacity for safe self-administration, and the complexity of the antimicrobial regimen. Clinical responsibility during OPAT and follow-up was shared between the referring team and the OPAT service, unless alternative arrangements had been agreed. Patients were reviewed regularly, and their progress was assessed during weekly virtual multidisciplinary meetings.

All patients referred for OPAT were assessed for suitability for self- or carer-administration and were accepted for S-OPAT only if they met predefined eligibility criteria. These included clinical stability, adequate manual dexterity and cognitive capacity to undertake safe self-administration, and a home environment supportive of treatment delivery. Additional requirements included willingness to engage in training, the ability to recognise and promptly report complications, and the absence of safeguarding or adherence concerns. Only individuals who successfully completed competency-based training, typically delivered over three days, and were judged to be safe and reliable to manage therapy independently were approved for this model of care. All S-OPAT patients were reviewed in person at least once a week for clinical assessment, blood monitoring, and vascular access care.

### 2.3. Data Collection

We reviewed the OPAT databases and hospital electronic health records. Extracted data included patient demographics, comorbidities, treatment indication, administered antimicrobial agents, duration of OPAT therapy (bed-days saved), method of OPAT delivery, vascular access device, OPAT outcome, complications, rehospitalisation, and reasons for rehospitalisation. Age (in years) and Charlson Comorbidity Index (CCI) scores [12] were determined at the start of OPAT. Chronic kidney disease was defined as an estimated glomerular filtration rate of <60 mL/min/1.73 m^2^ for at least three months, or evidence of kidney damage (e.g., structural abnormalities, significant albuminuria) despite an eGFR is ≥60 mL/min/1.73 m^2^ [13]. Peripheral vascular access devices included midline catheters, Leaderflex catheters, peripheral cannulas, and butterfly cannulas. Central venous access devices included peripherally inserted central catheters (PICCs), Hickman lines, and portacaths (implanted ports). All patient data were anonymised prior to analysis.

### 2.4. Outcomes

Clinical outcome—categorised as cure, improvement, or failure—was assessed at the completion of parenteral antimicrobial therapy according to the definitions outlined in the British Society for Antimicrobial Chemotherapy National Outcomes Registry System (NORS) (Supplementary Table S1) [14]. OPAT failure was defined as progression or lack of response of the infection, rehospitalisation, need for surgical intervention, or death occurring during the OPAT treatment. Adverse events (AEs) encompassed adverse drug reactions (ADRs) (i.e., events attributable to the administered antimicrobial agents, such as blood dyscrasias, diarrhoea, other gastrointestinal disturbances, rash, and hepatic or renal dysfunction), vascular access-related complications (including dressing allergy, catheter-related infection, line migration, occlusion, or thrombosis), and infusion (elastomeric) device-related complications (such as device leakage or malfunction). A 30-day unplanned hospitalisation was defined as any unplanned inpatient admission to an acute care hospital for any cause occurring during OPAT or within 30 days after completion of OPAT.

### 2.5. Statistical Analysis

The effects of the OPAT delivery models on the risk of each binary outcome endpoint of interest were compared using risk ratios (RRs) estimated from a modified Poisson regression model with a log-link function and a robust (“sandwich”) variance estimator [15,16]. OPAT episodes were the unit of analysis. Generalised estimating equations (GEE) with an exchangeable correlation structure were used to account for within-patient correlation among individuals with multiple OPAT episodes. To control the family-wise error rate across three comparisons (S-OPAT vs. H-OPAT, S-OPAT vs. C-OPAT, and C-OPAT vs. H-OPAT), pairwise *p*-values were adjusted using the Bonferroni method [17], applying an adjusted alpha level of 0.017 and calculating 98.3% confidence intervals (CIs) for the RRs. Omnibus *p*-values were also reported to provide an overall assessment of group differences.

A priori baseline covariates selected for inclusion as potential confounders or predictors in the regression models were patient characteristics (age, sex, comorbidities, and CCI) and treatment-related variables (infection type, antimicrobial class, concomitant IV antimicrobial therapy, type of vascular access device, and OPAT duration). Multicollinearity was assessed using variance inflation factors (Supplementary Table S2). To maintain parsimonious regression models and avoid multicollinearity, the CCI score was included as a covariate rather than each of its 19 individual component comorbidities. Antimicrobial class was also excluded due to high correlation with infection type. OPAT duration was considered an intermediate variable in the causal pathway between baseline covariates and outcomes and was therefore excluded from the regression models. Log-linearity of quantitative variables was examined using restricted cubic splines (Supplementary Figure S1). Age was retained as a continuous variable, whereas the CCI score was categorised into quartiles (0–1, 2, 3–4, and 5+).

The modified robust Poisson model was also used to explore risk factors for treatment failure, OPAT-related AEs, and 30-day unplanned hospitalisation among S-OPAT patients. In addition to the composite CCI score, the most common comorbid conditions—those present in at least 30 patients—were examined. Owing to the small number of outcome events in this subgroup, RRs were adjusted only for age and sex.

There were no missing data for any study variables. Stata 19 (StataCorp LLC, College Station, TX, USA) was used for data management and analysis. A two-tailed *p*-value < 0.05 was considered statistically significant.

## 3. Results

### 3.1. Cohort Characteristics

**Figure 1:**
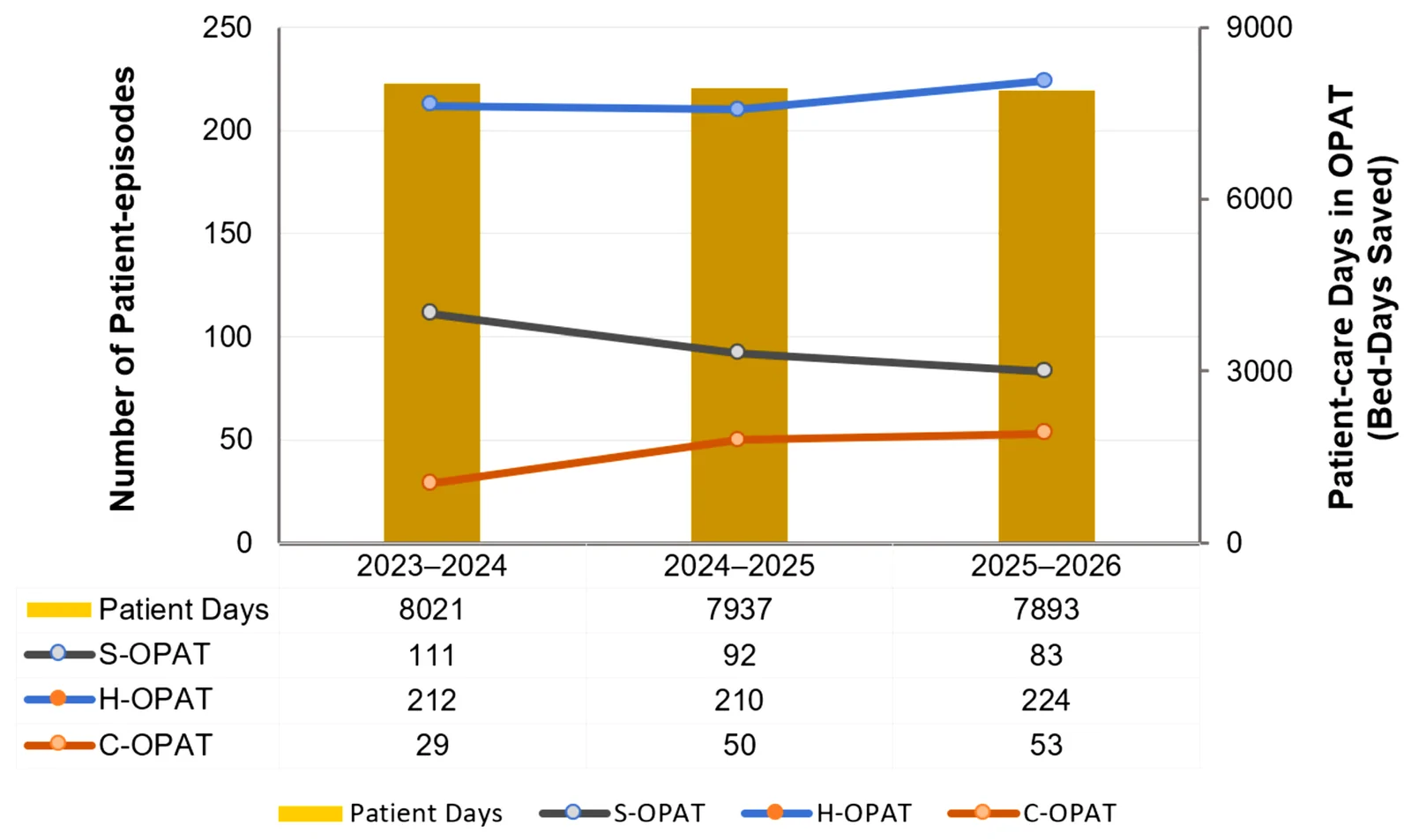
Annual OPAT activity from 2023 to 2026, showing the number of OPAT episodes and total patient-care days. C-OPAT, clinic-based OPAT; H-OPAT, healthcare-administered OPAT at home; OPAT, outpatient parenteral antimicrobial therapy; S-OPAT, self/carer-administered OPAT.

Over the three-year study period, 1064 OPAT episodes were delivered to 846 distinct patients. Annual OPAT activity remained stable throughout the study period (Figure 1).

Of all recorded episodes, 286 (26.9%) were delivered via S-OPAT, 646 (60.7%) were administered at home by community nurses (H-OPAT), and 132 (12.4%) were managed in the OPAT clinic (C-OPAT) (Table 1). Thirteen episodes involved hybrid OPAT, combining elements of the three delivery models. This included nine S-OPAT episodes requiring multiple daily doses of antimicrobials (meropenem, *n* = 6; ceftazidime, *n* = 3), in which one of the doses was administered at home by community nurses. Three further S-OPAT patients received two antimicrobial agents (daily ceftriaxone or meropenem together with teicoplanin) and attended the OPAT clinic on alternate days for teicoplanin (dosing adjusted according to renal function) administration. Approximately one-third (33.9%; 97/286) of S-OPAT episodes were administered by patients’ relatives or caregivers.

The median age of patients was 68 years (range, 17–97), and 38.3% (408/1064) were female (Table 1). The S-OPAT group had the lowest median age of 58 years (range 18–91), compared with 61 years in C-OPAT and 72 years in H-OPAT. The median CCI score of the cohort was 2 (range, 0–12), with diabetes as the most common underlying comorbidity. The most frequent indication for OPAT was bone and joint infection (43.2%). OPAT delivery accounted for 23,851 patient-care days (bed-days saved), with a median treatment duration of 14 days (IQR, 8–31) and an annual average of 7950 days. S-OPAT accounted for 29.5% (7032/23,851) of the total bed-days saved, equivalent to approximately six hospital beds freed per day.

The three most frequently administered antimicrobial agents were piperacillin/tazobactam (28.3%), teicoplanin (23.8%), and ertapenem (14.4%) (Supplementary Table S3). Overall, 5.6% (60/1064) of OPAT episodes involved co-administration of more than one parenteral antimicrobial agent, with only eight S-OPAT patients (2.8%) receiving concomitant IV therapy. Most patient episodes (82.0%, 873/1064) received antimicrobials via peripheral vascular access, predominantly midlines (99.4%; 868/873).

### 3.2. Clinical Outcomes

A total of 912 episodes (85.7%) met the NORS definition of cure or clinical improvement (Table 2) [14]. Treatment failure occurred in 152 episodes (14.3%), most commonly due to rehospitalisation for worsening of the treated infection (38.1%; 56/147) followed by new, non-OPAT-related events such as falls or decompensated heart failure (28.6%; 42/147) (Supplementary Table S4). OPAT failure was observed in 26 S-OPAT episodes (9.1%). One patient managed through H-OPAT died; this individual had metastatic colonic cancer and received palliative OPAT for intercurrent infective endocarditis [10]. The death was anticipated given the underlying disease trajectory.

AEs were documented in 174 patient-episodes (16.4%; 7.3 events per 1000 OPAT-days), most of which were vascular access–related (3.9 events per 1000 OPAT-days (Table 2). Three patients (0.3%) experienced both antimicrobial-related and vascular access–related AEs. Rash was the most frequent drug-related AE (33.3%, 28/84), occurring predominantly among H-OPAT recipients. Among vascular access-related complications, line occlusion was most common (48.4%; 45/93), followed by line migration (23.7%; 22/93) (Supplementary Table S5). No complications attributable to elastomeric devices—such as leakage or malfunction—were observed. Complications were recorded in 46 S-OPAT episodes (16.1%).

Unplanned 30-day hospitalisation occurred in 252 (23.7%) patient-episodes, with worsening or inadequate response to the underlying infection (44.4%; 112/252) being the most common reason, followed by non-OPAT-related causes (27.4%) and new infections (15.1%).

### 3.3. Comparison of OPAT Delivery Models

**Figure 2:**
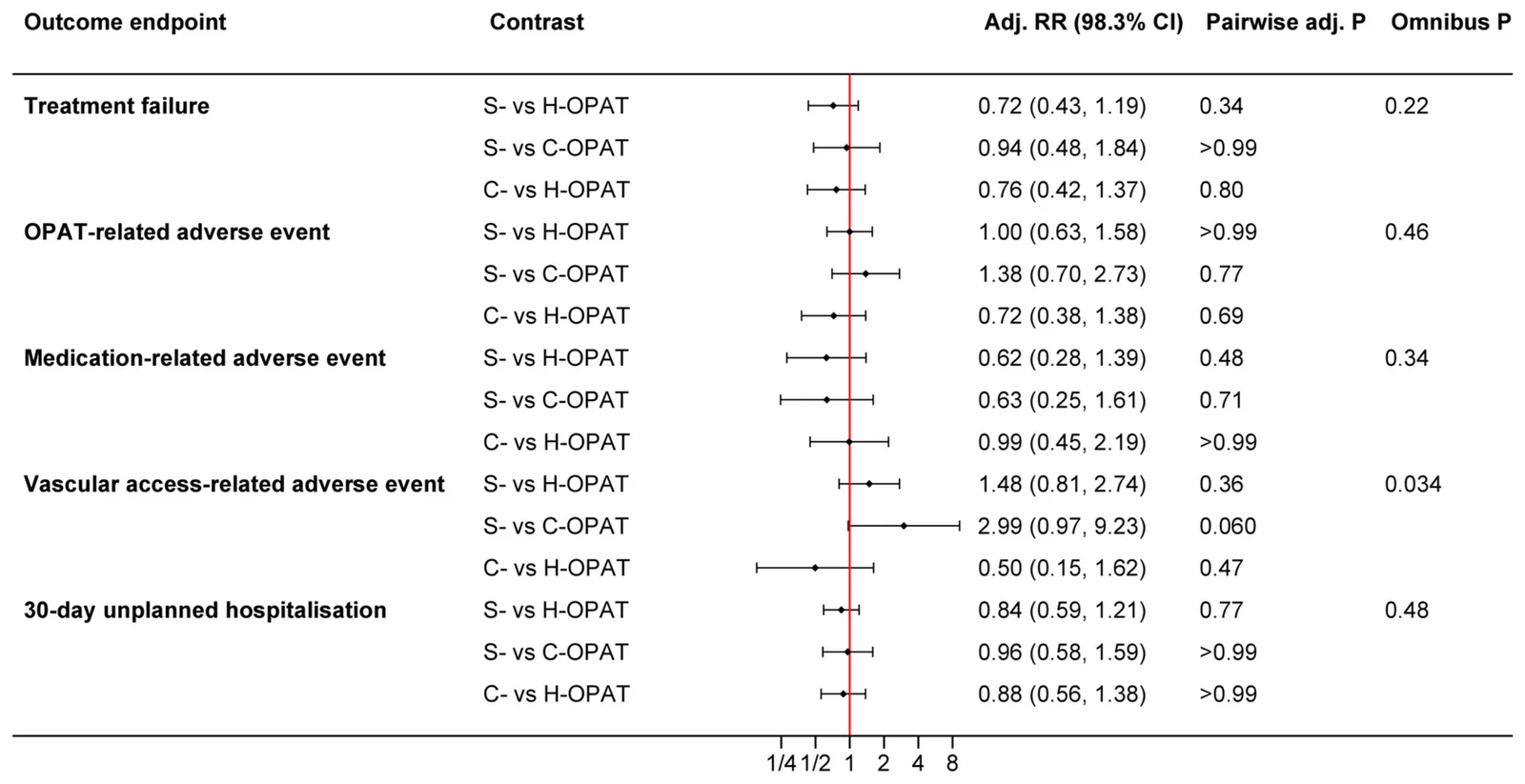
Comparison of clinical outcomes across different OPAT delivery models. Adj. *p*, adjusted *p*-value; Adj. RR, adjusted risk ratio; C-OPAT, clinic-based OPAT; CI, confidence interval; H-OPAT, healthcare-administered OPAT at home; OPAT, outpatient parenteral antimicrobial therapy; S-OPAT, self/carer-administered OPAT.

Significant differences in infection outcomes were observed across OPAT delivery models in univariate analysis (Table 2). S-OPAT demonstrated the highest proportion of successful outcomes, with 260 patient episodes (90.9%) achieving cure or clinical improvement, compared with 82.7% and 89.4% in H-OPAT and C-OPAT, respectively. However, after correcting for the differences in patient characteristics and treatment-related variables, pairwise comparisons showed no significant differences in treatment failure between S-OPAT and H-OPAT (adjusted RR, 0.72; 98.3% CI, 0.43–1.19) or between S-OPAT and C-OPAT (adjusted RR, 0.94; 98.3% CI, 0.48–1.84). Although lower relative risks were observed for S-OPAT than for H-OPAT and C-OPAT in the study sample, these differences were not statistically significant (Figure 2). For reference, the unadjusted RRs for the effects of the different OPAT delivery models on clinical outcomes are presented in Supplementary Figure S2.

OPAT-related risks were not significantly different across the OPAT delivery models (*p* = 0.46) (Figure 2). ADRs were likewise comparable, with no significant evidence of an overall difference (omnibus *p* = 0.34). However, vascular access–related complications differed significantly (*p* = 0.034). The highest incidence was observed in the S-OPAT group (11.5%), with an adjusted RR of 1.48 (98.3% CI, 0.81–2.74) compared with H-OPAT, and an adjusted RR of 2.99 (95% CI, 0.97–9.23) compared with C-OPAT.

Differences in 30-day unplanned hospitalisation across delivery models were also significant, with lower proportions in S-OPAT (19.6%) and C-OPAT (19.7%) compared with H-OPAT (26.3%) in the univariate analysis. However, the multivariable analyses did not show statistically significant evidence for overall or pairwise differences in the risk of unplanned hospitalisation between delivery models (omnibus *p* = 0.48; Figure 2), indicating that the crude differences were not sustained after accounting for relevant covariates.

### 3.4. Risk Factors for Treatment Failure, AEs, and Unplanned Hospitalisation in S-OPAT

Table 3 presents the results of the relative risk regression analyses, adjusted for age and sex, examining factors associated with poor outcomes among the 286 patient-episodes managed via S-OPAT. Older age (adjusted RR 1.04 per year increase; 95% CI, 1.01–1.07; *p* = 0.007), higher CCI score (adjusted RR per unit increase, 1.29; 95% CI, 1.12–1.49; *p* < 0.001), diabetes (adjusted RR, 3.12; 95% CI, 1.59–6.13; *p* < 0.001), peripheral vascular disease (adjusted RR, 2.32; 95% CI, 1.06–5.11; *p* = 0.036), and use of central venous access devices (adjusted RR, 2.43; 95% CI, 1.28–4.63; *p* = 0.007) were significantly associated with treatment failure. No statistically significant associations were observed with sex, other comorbidities, OPAT indication, antimicrobial class, dosing frequency, or treatment administrator (patient versus relative/caregiver).

Higher CCI score (adjusted RR, 1.25 per unit increase; 95% CI, 1.12–1.40; *p* < 0.001) and diabetes (adjusted RR, 2.64; 95% CI, 1.59–4.39; *p* < 0.001) were also associated with an increased risk of 30-day unplanned hospitalisation. Central venous access showed an increased, albeit not statistically significant, risk of hospitalisation (adjusted RR, 1.71; 95% CI, 0.98–2.98; *p* = 0.058).

Regarding OPAT-related AEs, chronic pulmonary disease (adjusted RR, 0.39; 95% CI, 0.18–0.86; *p* = 0.019) and respiratory infections (adjusted RR, 0.33; 95% CI, 0.13–0.88; *p* = 0.027), compared with other indications, were associated with lower risks of complications.

For reference, adjusted RRs derived from GEE-based robust Poisson models are presented in Supplementary Tables S6–S10.

## 4. Discussion

In this retrospective cohort study of 1064 OPAT episodes, we compared three commonly used delivery models—S-OPAT, H-OPAT, and C-OPAT—and examined patient- and treatment-related factors associated with poor clinical outcomes among those undertaking self-administration. Overall, S-OPAT was associated with a high rate of treatment success, with 91% of episodes resulting in cure or clinical improvement. Although S-OPAT had the most favourable crude treatment outcomes and the lowest crude rate of 30-day unplanned hospitalisation, these differences were not maintained after adjustment for potential confounding factors and clustering of multiple treatment episodes within individual patients. Within the S-OPAT group, older age, diabetes, higher comorbidity burden, peripheral vascular disease, and central venous access were associated with treatment failure, while diabetes and higher comorbidity burden were associated with 30-day unplanned hospitalisation.

The absence of a significant difference in adjusted treatment failure and 30-day hospitalisation between S-OPAT and either H-OPAT or C-OPAT in our cohort is consistent with previous studies [18,19,20] and adds to the growing body of evidence that appropriately selected patients can safely and effectively self-administer IV antimicrobial therapy in the community within a structured OPAT programme [7]. The overall cure or clinical improvement rate of 86% in our cohort is also consistent with prior OPAT studies reporting treatment success rates above 80% [21]. Importantly, the higher crude success rate observed with S-OPAT should not be interpreted as evidence that self-administration is intrinsically more effective than healthcare professional-supported models. Patients were not randomly allocated to delivery models, and, consistent with previous studies [18,19,20], those selected for S-OPAT were younger and had a lower comorbidity burden than patients receiving H-OPAT. The attenuation of differences following adjustment therefore suggests that patient selection and underlying case-mix, rather than the delivery model itself, are key determinants of clinical outcome. This finding reinforces the importance of considering OPAT delivery as an individualised clinical decision rather than assuming that one delivery model is universally superior.

The overall frequency of OPAT-related AEs was 16%, comparable to previous OPAT cohorts [19,21], further supporting the favourable safety profile of OPAT programmes. However, we observed a pattern similar to that reported by Sriskandarajah et al. in a paediatric OPAT cohort [22]. Specifically, vascular access–related AEs were more frequent among patients who self-administered therapy, whereas ADRs occurred more commonly in nurse-administered OPAT. Nevertheless, medication-related AEs did not differ significantly between delivery models. Although vascular access–related complications, predominantly line occlusions, were more common in S-OPAT episodes, the adjusted pairwise comparisons were not consistently statistically significant. 

An increased incidence of vascular-access–related complications among patients receiving S-OPAT has also been previously described [19,23]. For example, Underwood et al. examined drug- and vascular access–related AEs in a large UK OPAT centre and found that self-administration was associated with a higher rate of catheter-related complications compared with nurse-administered or mixed models [23]. In our cohort, one possible explanation is that patients who self-administer may have been more likely to recognise and report vascular access problems promptly, given that such complications directly interfere with their ability to continue therapy. Conversely, complications occurring among patients managed through healthcare professional–supported models (i.e., H-OPAT and C-OPAT) may be recognised during routine nursing encounters and may not always be recorded in the same manner. Therefore, differences in ascertainment and reporting may contribute to the observed variation between delivery models as much as true differences in event rates. Nonetheless, we are reviewing our AE-reporting processes to improve the consistency of surveillance across all OPAT delivery pathways. In parallel, we are strengthening patient education on vascular access care and troubleshooting strategies to support early recognition and management of complications and reduce the risk of future vascular access–related events.

The analysis of the S-OPAT subgroup identified several characteristics associated with treatment failure. Notably, S-OPAT delivery characteristics—including patient- or relative-administered therapy, antimicrobial class, and dosing frequency—were not associated with poor outcomes. Instead, increasing age was associated with a 4% increase in the relative risk of treatment failure for each additional year of age, while underlying comorbidities and central venous access were also independently associated with treatment failure. These findings are broadly consistent with previous OPAT studies demonstrating poorer outcomes among older patients and those with greater comorbidity [7,24]. The association with age is unlikely to indicate that chronological age itself should be considered a contraindication to S-OPAT. Rather, older age may act as a marker for frailty, functional impairment, multimorbidity, and reduced physiological reserve [25,26]. Assessment of functional and cognitive capacity, manual dexterity, social support, and ability to recognise and respond to complications may therefore provide more clinically useful information than age alone.

Associations of higher CCI scores and diabetes with both treatment failure and 30-day unplanned hospitalisation, as seen in the S-OPAT group in this study, have also been reported in conventional OPAT cohorts [7,20,27,28,29,30,31,32]. Individuals with diabetes frequently have multiple coexisting conditions, which may collectively contribute to increased vulnerability to adverse outcomes [33]. More broadly, patients with higher comorbidity burden often experience increased healthcare utilisation, altered pharmacokinetics, reduced physiological reserve, and greater challenges in adhering to complex treatment regimens [34,35,36]. Collectively, these findings suggest that comorbidity should form an important component of S-OPAT risk assessment tools and may help identify patients who would benefit from enhanced monitoring or an alternative OPAT delivery model. Importantly, however, the presence of comorbidity should not automatically exclude patients from S-OPAT. Instead, these findings support a risk-stratified approach in which the intensity of monitoring and level of clinical support are tailored to individual patient characteristics.

Central venous access was associated with more than a two-fold increased risk of S-OPAT treatment failure. The mechanism underlying this association is uncertain. It is likely attributed, at least in part, to confounding by indication, as patients requiring central access typically have prolonged treatment courses, deep-seated or complex infections, or require antimicrobials unsuitable for peripheral administration. Furthermore, central venous devices carry an inherent risk of catheter-related AEs, including bloodstream infection and mechanical dysfunction, which may also contribute to poorer clinical outcomes [37,38,39]. In our study, however, central access was not significantly associated with increased risk of overall OPAT-related complications and was only borderline associated with 30-day hospitalisation. The observed association with treatment failure should therefore be interpreted cautiously and should not be regarded as evidence of a causal effect of central venous access itself. Nevertheless, optimising vascular access device selection and adherence to best practices in catheter care remain important modifiable strategies to enhance safety within OPAT services. Of note, we found no patient- or treatment-related variables to be associated with increased risk of OPAT-related complications, suggesting that the occurrence of complications in this cohort may have been driven by factors not captured in the present analysis.

An important finding was that treatment administrator (patient versus relative/caregiver) within the S-OPAT group was not associated with treatment failure, adverse events, or unplanned hospitalisation. Approximately one-third of S-OPAT episodes in our cohort were administered by family members or caregivers, suggesting that appropriately trained caregivers may extend the applicability of S-OPAT to patients who might otherwise be unable to undertake treatment independently. Caregiver involvement may also support administration of multiple daily doses, reinforce treatment instructions, facilitate early recognition of complications, and provide additional assistance with treatment monitoring.

The concept of patients safely self-administering parenteral antimicrobials at home would, until recently, have been viewed with considerable scepticism. Our study provides real-world evidence supporting S-OPAT as a safe, feasible, and potentially resource-sparing component of modern OPAT services, adding to the growing literature in this area [7]. It further advances the evidence base by defining S-OPAT’s comparative performance and identifying patients at increased risk of adverse outcomes through direct comparison of the three commonly used OPAT delivery models within the same service—an approach that offers greater analytical clarity than studies restricted to a single model or reliant on pooled traditional OPAT comparators [7,18,19,20,22,23]. The success of S-OPAT in this study likely reflects the careful patient selection and structured training delivered by OPAT nurses, weekly in-person reviews, and rapid access to specialist advice. These components are essential for safe implementation and should be considered core requirements for any service adopting S-OPAT.

Overall, our findings may inform patient-selection criteria, guide service development, and strengthen the case for broader expansion of patient-centred care. The identification of risk factors for S-OPAT failure provides a basis for moving towards risk-stratified rather than binary selection for S-OPAT, thereby enabling more targeted decision-making while ensuring that patients are matched to the most appropriate model of care.

Beyond favourable clinical outcomes, S-OPAT confers important operational advantages [5,40,41,42,43]. Patients undertaking self-administration required fewer clinic visits and less community nursing input, thereby alleviating pressure on ambulatory care and district nursing services (Table 4). This is particularly relevant given rising OPAT demand, workforce constraints, and the imperative to optimise resource utilisation across healthcare systems [3]. Accordingly, and in line with Stoorvagel et al. [4], we advocate that OPAT services offer S-OPAT to all suitable patients and/or their caregivers, with flexibility to trial and tailor the approach to individual needs.

By enabling antimicrobial regimens requiring multiple daily dosing—often difficult to deliver in H-OPAT or C-OPAT models—S-OPAT expands therapeutic options for out-of-hospital care and supports earlier hospital discharge. For patients requiring recurrent courses of parenteral antimicrobial therapy, such as those experiencing frequent infective exacerbations of bronchiectasis, S-OPAT may reduce the burden of repeated hospital attendances while maintaining timely access to treatment. Once appropriately trained, such patients can initiate therapy promptly at home or in outpatient settings with minimal need for repeated and/or extensive in-hospital instruction.

In addition to its clinical and operational implications, S-OPAT may have relevant antimicrobial stewardship (AMS) implications. Although S-OPAT is primarily a delivery model rather than a prescribing intervention, it may support stewardship by enabling selected patients to complete parenteral therapy in the community under continued OPAT specialist oversight. Nevertheless, the convenience of outpatient administration should not drive unnecessary prolongation of IV or broad-spectrum therapy. Antimicrobial indication, spectrum, dose, duration, and opportunities for de-escalation or IV-to-oral switch (IVOST) should therefore remain subject to regular multidisciplinary review across all OPAT pathways. Although our finding that antimicrobial class, dosing frequency, and treatment duration were not associated with S-OPAT failure or adverse outcomes is reassuring, it does not demonstrate a direct stewardship benefit.

Despite its benefits, S-OPAT is not suitable for all patients. Individuals with significant comorbidities, complex infections, safeguarding concerns, or limited capacity for self-management may require alternative delivery models to ensure safe and effective treatment. S-OPAT may also be less appropriate for short treatment courses (e.g., for cellulitis), as the required training can take several days. These considerations emphasise the need for a flexible, multimodal OPAT service capable of tailoring delivery to individual patient needs. While S-OPAT can reduce healthcare utilisation, it transfers responsibility to patients and caregivers. Ensuring adequate support, clearly defined roles, effective communication, accessible escalation pathways, and ongoing clinical oversight is therefore essential to maintaining safety and patient confidence [44]. Where treatment burden for patients or caregivers is a concern [45,46], a hybrid model that combines self-administration with elements of other OPAT delivery approaches—used in 13 patients in our cohort—may help alleviate these challenges. The use of hybrid pathways in our cohort supports conceptualising OPAT delivery as a continuum rather than a set of discrete, mutually exclusive models. Such flexibility is particularly valuable when antimicrobial dosing requirements or patient capability do not align neatly with a single delivery modality. Additionally, the use of pre-filled disposable elastomeric devices for continuous infusion of suitable antimicrobials (subject to stability), such as flucloxacillin or piperacillin/tazobactam, may further reduce treatment complexity and patient burden by eliminating the need for drug preparation, reconstitution, or complex programming [9,11,47]. Moreover, telemedicine approaches—including video consultations—can strengthen clinical oversight and support the safer and more efficient delivery of S-OPAT by enabling virtual line assessments, remote symptom evaluation, and early detection of complications [48]. Lastly, a structured educational toolkit has the potential to reduce treatment burden by streamlining essential tasks, clarifying expectations, and improving confidence in self-management [44,46].

Our study has several strengths, including the use of real-world data from a large regional OPAT service, comprehensive characterisation of different delivery models, and systematic evaluation of risk factors associated with poor outcomes. Furthermore, the application of GEE accounted for correlations arising from multiple treatment episodes within individual patients, thereby providing more robust estimates of comparative outcomes. Nevertheless, several limitations should be considered. The single-centre design may limit generalisability, and selection bias related to S-OPAT eligibility criteria is possible. Patients were not randomly assigned to delivery models, and unmeasured confounding factors may have influenced both treatment allocation and clinical outcomes. Reporting bias cannot be excluded, and the observational design precludes causal inference. The relatively small numbers of specific AEs, particularly in the clinic-based group, led to wide CIs and reduced statistical precision for some pairwise comparisons. In addition, some potentially relevant variables, including detailed measures of infection severity, adherence to therapy, microbiological data, and socioeconomic factors, were not available for analysis. Future research should seek to validate our findings in larger, multicentre cohorts and explore patient-reported outcomes, cost-effectiveness, and the broader impact of S-OPAT on AMS and long-term service sustainability across diverse healthcare settings.

## 5. Conclusions

Self-administered OPAT was found to be a safe and effective alternative to traditional delivery models in appropriately selected patients. As demand for OPAT continues to increase, integrating S-OPAT within a flexible, multimodal service model may provide a sustainable approach to expanding capacity while preserving the quality and safety of care.

## Figures and Tables

**Table 1:** Baseline clinical and treatment characteristics according to OPAT delivery model.

Characteristics	Total (*N* = 1064)	S-OPAT (*n* = 286)	H-OPAT (*n* = 646)	C-OPAT (*n* = 132)
Age (years), median (IQR)	68 (57–76)	58 (48–70)	72 (62–79)	61 (48–68)
Male sex, *n* (%)	656 (61.7)	177 (61.9)	403 (62.4)	76 (57.6)
Charlson comorbidity score, median (IQR)	2 (1–4)	1 (0–3)	2 (1–4)	1 (0–3)
Chronic comorbid conditions				
Cerebrovascular disease	98 (9.2)	15 (5.2)	77 (11.9)	6 (4.5)
Chronic pulmonary disease	254 (23.9)	91 (31.8)	144 (22.3)	19 (14.4)
Connective tissue disease	85 (8.0)	26 (9.1)	54 (8.4)	5 (3.8)
Dementia	30 (2.8)	3 (1.0)	25 (3.9)	2 (1.5)
Diabetes with complications	274 (25.8)	57 (19.9)	194 (30.0)	23 (17.4)
Diabetes without complication	125 (11.7)	29 (10.1)	85 (13.2)	11 (8.3)
Heart failure	160 (15.0)	15 (5.2)	129 (20.0)	16 (12.1)
Hemiplegia or paraplegia	12 (1.1)	-	11 (1.7)	1 (0.8)
Leukaemia	16 (1.5)	4 (1.4)	9 (1.4)	3 (2.3)
Lymphoma or myeloma	13 (1.2)	2 (0.7)	9 (1.4)	2 (1.5)
Metastatic solid tumour	24 (2.3)	3 (1.0)	14 (2.2)	7 (5.3)
Mild liver disease	35 (3.3)	8 (2.8)	18 (2.8)	9 (6.8)
Moderate or severe liver disease	32 (3.0)	14 (4.9)	13 (2.0)	5 (3.8)
Moderate or severe renal disease	230 (21.6)	40 (14.0)	167 (25.9)	23 (17.4)
Myocardial infarction	128 (12.0)	24 (8.4)	94 (14.6)	10 (7.6)
Peptic ulcer disease	16 (1.5)	2 (0.7)	13 (2.0)	1 (0.8)
Peripheral vascular disease	151 (14.2)	37 (12.9)	104 (16.1)	10 (7.6)
Tumour without metastasis	54 (5.1)	14 (4.9)	34 (5.3)	6 (4.5)
Indication for OPAT, *n* (%)				
Bacteraemia	9 (0.8)	2 (0.7)	5 (0.8)	2 (1.5)
Bone and joint infection ^a^	460 (43.2)	99 (34.6)	305 (47.2)	56 (42.4)
CNS infection	17 (1.6)	8 (2.8)	4 (0.6)	5 (3.8)
Endovascular infection ^b^	111 (10.4)	13 (4.5)	81 (12.5)	17 (12.9)
ENT infection ^c^	66 (6.2)	26 (9.1)	37 (5.7)	3 (2.8)
Hepatobiliary infection	24 (2.3)	5 (1.7)	18 (2.8)	1 (0.8)
Intra-abdominal infection ^d^	14 (1.3)	6 (2.1)	5 (0.8)	3 (2.3)
Invasive fungal infection	2 (0.2)	-	-	2 (1.5)
Line infection	3 (0.3)	-	2 (0.3)	1 (0.8)
Respiratory infection ^e^	206 (19.4)	79 (27.6)	114 (17.6)	13 (9.8)
Skin and soft tissue infection	38 (3.6)	7 (2.4)	21 (3.3)	10 (7.6)
Spinal infection ^f^	56 (5.3)	15 (5.2)	33 (5.1)	8 (6.1)
Urinary tract infection ^g^	58 (5.5)	26 (9.1)	21 (3.3)	11 (8.3)
IV Antimicrobial agent, *n* (%) ^h^				
Antifungal	3 (0.3)	1 (0.3)	-	2 (1.4)
Carbapenem	188 (16.7)	60 (20.4)	106 (15.4)	22 (15.6)
Cephalosporin	176 (15.7)	54 (18.4)	100 (14.5)	22 (15.6)
Glycopeptide	271 (24.1)	16 (5.4)	200 (29.0)	55 (39.0)
Penicillin	457 (40.7)	161 (54.8)	264 (38.3)	32 (22.7)
Others	29 (2.6)	2 (0.7)	19 (2.8)	8 (5.7)
Concomitant IV antimicrobial therapy, *n* (%)	60 (5.6)	8 (2.8)	43 (6.7)	9 (6.8)
Type of vascular access, *n* (%)				
Central line	191 (18.0)	61 (21.3)	111 (17.2)	19 (14.4)
Peripheral access	873 (82.0)	225 (78.7)	535 (82.8)	113 (85.6)
Duration of OPAT, days, median (IQR)	14 (8–31)	14 (9–31)	15 (8–32)	12 (6–29)

Data are presented as *n* (%) unless otherwise indicated. C-OPAT, clinic-based OPAT; CNS, central nervous system; ENT, ear, nose, and throat; H-OPAT, healthcare-administered OPAT at home; IQR, interquartile range; IV; intravenous; OPAT, outpatient parenteral antimicrobial therapy; S-OPAT, self/carer-administered OPAT. ^a^ Bone and joint infection: spinal infection excluded; includes septic arthritis, prosthetic joint infection, non-diabetic osteomyelitis, and diabetic foot osteomyelitis. ^b^ Endovascular infection: includes infective endocarditis and vascular graft infection. ^c^ ENT infection: predominantly necrotising otitis externa. ^d^ Intra-abdominal infection: hepatobiliary infection excluded; predominantly intrabdominal collections. ^e^ Respiratory infection: mainly infective exacerbations of bronchiectasis and chronic obstructive pulmonary disease. ^f^ Spinal infection: includes discitis, vertebral osteomyelitis, and epidural abscess. ^g^ Urinary tract infection: mainly complex urinary tract infection. ^h^ Some patients received more than one antimicrobial agent; therefore, the total number of antimicrobial agents exceeds the number of patient episodes. Percentages are calculated using the total number of antimicrobial agents as the denominator. Antifungal agents comprised anidulafungin (*n* = 1), caspofungin (*n* = 1), and rezafungin (*n* = 1). Carbapenems comprised ertapenem (*n* = 162) and meropenem (*n* = 26). Cephalosporins comprised ceftazidime (*n* = 16), ceftriaxone (*n* = 158), and cefuroxime (*n* = 2). Glycopeptides comprised dalbavancin (*n* = 4) and teicoplanin (*n* = 267). Penicillins comprised flucloxacillin (*n* = 139) and piperacillin/tazobactam (*n* = 318). Other agents included daptomycin (*n* = 28) and tigecycline (*n* = 1). A full antimicrobial breakdown is provided in Table S3.

**Table 2:** Clinical outcomes according to OPAT delivery model.

Variable	Total (*N* = 1064)	S-OPAT (*n* = 286)	H-OPAT (*n* = 646)	C-OPAT (*n* = 132)	*p* Value ^a^
Infection outcome, *n* (%)					
Cured or improved	912 (85.7%)	260 (90.9%)	534 (82.7%)	118 (89.4%)	0.003
Failure	152 (14.3%)	26 (9.1%)	112 (17.3%)	14 (10.6%)	
OPAT-related adverse event, *n* (%); events per 1000 OPAT-days ^b^	174 (16.4%); 7.3/1000 days	46 (16.1%); 6.5/1000 days	111 (17.2%); 7.8/1000 days	17 (12.9%); 6.8/1000 days	0.45
Medication-related, *n* (%); events per 1000 OPAT-days	84 (7.9%); 3.5/1000 days	15 (5.2%); 2.1/1000 days	58 (9.0%); 4.1/1000 days	11 (8.3%); 4.4/1000 days	0.18
Vascular access-related, *n* (%); events per 1000 OPAT-days	93 (8.7%); 3.9/1000 days	33 (11.5%); 4.7/1000 days	54 (8.4%); 3.8/1000 days	6 (4.5%); 2.4/1000 days	0.028
30-day unplanned hospitalisation, *n* (%)	252 (23.7%)	56 (19.6%)	170 (26.3%)	26 (19.7%)	0.041
Reason for unplanned hospitalisation, *n* (%)					
Adverse drug reaction	22 (8.7%)	2 (3.6%)	18 (10.6%)	2 (7.7%)	0.022
IV line-related complication	11 (4.4%)	6 (10.7%)	4 (2.4%)	1 (3.8%)	
New infection	38 (15.1%)	5 (8.9%)	32 (18.8%)	1 (3.8%)	
Non-OPAT related	69 (27.4%)	13 (23.2%)	50 (29.4%)	6 (23.1%)	
Worsening of infection/no improvement	112 (44.4%)	30 (53.6%)	66 (38.8%)	16 (61.5%)	

C-OPAT, clinic-based OPAT; H-OPAT, healthcare-administered OPAT at home; OPAT, IV; intravenous; OPAT, outpatient parenteral antimicrobial therapy; S-OPAT, self/carer-administered OPAT. ^a^ *p*-values refer to the crude comparison of proportions across OPAT delivery models without accounting for other variables. Generalised estimating equations were used to account for correlations between patients with multiple OPAT episodes. ^b^ Some patient episodes had more than one adverse event.

**Table 3:** Exploratory analysis of risk factors for adverse clinical outcomes among 286 self-/carer-administered OPAT episodes.

Risk Factor	Treatment Failure		OPAT-Related Complication		30-Day Unplanned Hospitalisation	
	Adj. RR (95% CI)	*p*-Value	Adj. RR (95% CI)	*p*-Value	Adj. RR (95% CI)	*p*-Value
Male sex	0.67 (0.31–1.44)	0.308	0.60 (0.33–1.07)	0.086	0.64 (0.37–1.10)	0.104
Age (per year increase)	1.04 (1.01–1.07)	0.007	1.01 (0.99–1.03)	0.425	1.01 (0.99–1.03)	0.174
Diabetes mellitus	3.12 (1.59–6.13)	<0.001	1.55 (0.86–2.79)	0.142	2.64 (1.59–4.39)	<0.001
CCI score (per unit increase)	1.29 (1.12–1.49)	<0.001	1.09 (0.95–1.25)	0.227	1.25 (1.12–1.40)	<0.001
Common comorbidities						
Chronic pulmonary disease	0.60 (0.27–1.34)	0.215	0.39 (0.18–0.86)	0.019	1.16 (0.66–2.05)	0.600
Moderate or severe renal disease	1.29 (0.53–3.16)	0.576	0.72 (0.32–1.64)	0.436	1.40 (0.68–2.88)	0.363
Peripheral vascular disease	2.32 (1.06–5.11)	0.036	1.55 (0.71–3.36)	0.269	1.61 (0.85–3.08)	0.145
Common indications						
Bone and joint infection	1.35 (0.63–2.92)	0.439	1.15 (0.65–2.05)	0.628	1.23 (0.67–2.26)	0.514
Respiratory infection	0.46 (0.17–1.26)	0.132	0.33 (0.13–0.88)	0.027	1.14 (0.57–2.28)	0.705
Others	1.00		1.00		1.00	
Common antimicrobials						
Penicillin	1.00		1.00		1.00	
Cephalosporin	1.87 (0.78–4.47)	0.160	1.01 (0.48–2.12)	0.983	1.16 (0.61–2.19)	0.650
Carbapenem	1.08 (0.40–2.92)	0.884	1.23 (0.62–2.44)	0.551	1.12 (0.62–2.01)	0.716
Others	1.57 (0.32–7.65)	0.573	1.36 (0.47–3.93)	0.574	0.84 (0.22–3.23)	0.798
Type of vascular access						
Peripheral access	1.00		1.00		1.00	
Central line	2.43 (1.28–4.63)	0.007	1.14 (0.57–2.26)	0.707	1.71 (0.98–2.98)	0.058
Dosing frequency						
Daily dosing	1.00		1.00		1.00	
Continuous Infusion	0.58 (0.29–1.15)	0.119	1.11 (0.64–1.93)	0.708	0.85 (0.52–1.40)	0.520
Individual delivering therapy						
Patient	1.00		1.00		1.00	
Relative	1.88 (0.84–4.22)	0.124	1.42 (0.81–2.48)	0.221	1.46 (0.85–2.51)	0.172

Adj. RR, adjusted risk ratio derived from a robust Poisson regression model adjusting for age and sex.; CCI, Charlson comorbidity index; CI, confidence interval.

**Table 4:** Comparison of outpatient parenteral antimicrobial therapy (OPAT) delivery models.

Model	Pros	Cons
C-OPAT	Allows regular in-person monitoring and supervised administration Suitable for patients needing closer oversight but not hospitalisation Relatively efficient in terms of staff time Reduces nursing travel costs	Travel requirements for patients which may be difficult for those with mobility or transport challenges Least convenient for patients Higher staffing and infrastructure requirements Clinic capacity can become a bottleneck Not ideal for multiple daily dosing Least favourable in terms of carbon footprint
H-OPAT	Provides high level of supervision in the patient’s home Minimises patient travel Most suitable for patients with mobility limitations Often most preferred by patients	Resource-intensive: high staffing and travel costs Safety of staff may present a concern Challenging where community nursing is not readily available Not ideal for multiple daily dosing Scheduling constraints may reduce flexibility for patients Environmental impact from travel and equipment use Limited scalability compared with S-OPAT
S-OPAT	Enhances patient autonomy and flexibility Can improve patient satisfaction and convenience Reduces need for healthcare visits, improving service capacity Often more resource-efficient and cost-effective Ideal for patients requiring multiple daily dosing, prolonged, or multiple treatment courses Most favourable in terms of carbon footprint	Requires robust training, governance, and competency assessment Patients must possess dexterity and training to perform necessary tasks Potential burden on patients and/or caregivers Not suitable for patients with complex comorbidities, safeguarding concerns, or limited ability to self-manage Potential clinical risks including administration errors and non-compliance due to less supervision

C-OPAT, clinic-based OPAT; H-OPAT, healthcare-administered OPAT at home; OPAT, outpatient parenteral antimicrobial therapy; S-OPAT, self/carer-administered OPAT.

## Data Availability

The data presented in this study are available on request from the corresponding author due to privacy.

## Supplementary Materials

The following supporting information can be downloaded at: https://www.mdpi.com/article/10.3390/pathogens15090981/s1, Table S1: BSAC NORS definitions of OPAT outcomes; Table S2: Multicollinearity assessment among baseline covariates, prior to performing regression analysis; Table S3: Intravenous antimicrobial agents administered during OPAT; Table S4: Description of clinical outcomes; Table S5: Frequency of adverse events; Table S6: Relative risk regression analysis of the association between OPAT delivery models and treatment failure in 1064 OPAT episodes from 846 distinct patients; Table S7: Relative risk regression analysis of the association between OPAT delivery models and risk of any OPAT-related adverse event in 1064 OPAT episodes from 846 distinct patients; Table S8: Relative risk regression analysis of the association between OPAT delivery models and risk of medication-related adverse events in 1064 OPAT episodes from 846 distinct patients; Table S9: Relative risk regression analysis of the association between OPAT delivery models and risk of vascular access-related adverse events in 1064 OPAT episodes from 846 distinct patients; Table S10: Relative risk regression analysis of the association between OPAT delivery models and 30-day unplanned hospitalisation in 1064 OPAT episodes from 846 distinct patients; Figure S1: Graphical assessment of the linearity of the relationship between clinical outcomes (treatment failure, OPAT-related adverse events, and 30-day unplanned hospitalisation) and continuous covariates (age and Charlson comorbidity index score); Figure S2: Comparison of crude (unadjusted) clinical outcomes across OPAT delivery models.

## Abbreviations

The following abbreviations are used in this manuscript:

ADRs	Adverse drug reactions
AEs	Adverse events
AMS	Antimicrobial stewardship
aRR	Adjusted relative risk
C-OPAT	Clinic-based outpatient parenteral antimicrobial therapy
CCI	Charlson Comorbidity Index
CI	Confidence interval
GEE	Generalised estimating equations
H-OPAT	Healthcare professional home-administered outpatient parenteral antimicrobial therapy
IV	Intravenous
IVOST	IV-to-oral switch
NORS	National Outcomes Registry System
OPAT	Outpatient parenteral antimicrobial therapy
PICC	Peripherally inserted central catheter
RR	Risk ratio
S-OPAT	Self-administered outpatient parenteral antimicrobial therapy
UHDB	University Hospitals of Derby and Burton
UK	United Kingdom
