# Supplementary material for: Comparing Self-Administered Outpatient Parenteral Antimicrobial Therapy (S-OPAT) with Alternative Delivery Models: Risk Factors for Treatment Failure and Complications in a UK Cohort
Source: Pathogens. 2026 Sep 15;15(9):981. doi: 10.3390/pathogens15090981 (PMC13610381; doi:10.3390/pathogens15090981)
Supplement: Supplementary file 1 [file pathogens-15-00981-s001.zip › pathogens-4547743-supplementary.pdf]

Comparing Self-Administered Outpatient Parenteral Antimicrobial Therapy (S-OPAT) with Alternative Delivery Models: Risk Factors for Treatment Failure and Complications in a UK Cohort

SUPPLEMENTARY DATA

Supplementary Table S1. BSAC NORS definitions of OPAT outcomes

**Infection outcomes**	
Cure	Completed OPAT therapy +/- oral step down for defined duration with resolution of infection and no requirement for long-term antibiotic therapy (usually relates to less severe infections e.g. SSTI, UTI unless prosthetic material removed).
Improved	i. Completed OPAT therapy +/- oral step down with partial resolution of infection but need for further follow up OR<br>ii. Completed OPAT therapy but required escalation of antimicrobial therapy during OPAT (without admission) +/- oral step down with ultimate cure or partial improvement (as above) e.g. osteomyelitis, any infections where prosthetic material has not been removed.
Failure	Progression or non-response of infection despite OPAT, required admission, surgical intervention or death for any reason.
**OPAT outcomes**	
Success	Completed therapy in OPAT with no change in antimicrobial agent, no adverse events, cure or improvement of infection and no readmission
Parial Success	Completed therapy in OPAT with either change in antimicrobial agent or adverse event not requiring admission
Failure of OPAT	Readmitted due to infection worsening or due to adverse event. Death due to any cause during OPAT
Indeterminate	Readmission due to unrelated event e.g. chest pain

BSAC, British Society for Antimicrobial Chemotherapy; NORS, National Outcomes Registry System; OPAT, outpatient parenteral antimicrobial therapy; SSTI, skin and soft tissue infection; UTI, urinary tract infection.

Supplementary Table S2. Multicollinearity assessment among baseline covariates, prior to performing regression analysis

**Variable**	**VIF** (full covariate list)	**VIF** (excluding highly collinear variables)
CCI score	239.81	excluded
Metastatic solid tumor	40.02	1.05
Diabetes with complications	38.68	1.80
Moderate or severe renal disease	34.77	1.35
Moderate or severe liver disease	13.86	1.06
Antimicrobial class: Penicillin	12.65	excluded
Chronic pulmonary disease	11.50	3.01
Tumour without metastasis	10.85	1.06
Antimicrobial class: Glycopeptide	8.46	excluded
Heart failure	7.95	1.33
Antimicrobial class: Carbapenem	7.38	excluded
Antimicrobial class: Cephalosporin	7.20	excluded
Peripheral vascular disease	7.20	1.30
Myocardial infarction	6.32	1.23
Diabetes without complication	6.06	1.18
OPAT indication: Respiratory infection	5.92	5.47
OPAT indication: Bone and joint infection	5.21	4.95
Cerebrovascular disease	5.18	1.13
Hemiplegia or paraplegia	4.66	1.03
Connective tissue disease	4.48	1.09
Leukaemia	3.62	1.12
Concomitant IV antimicrobial therapy	3.54	1.12
Lymphoma or myeloma	3.39	1.03
Mild liver disease	2.78	1.06
OPAT Delivery Model: Self-administered	2.68	2.50
OPAT Delivery Model: Healthcare-administered	2.66	2.60
OPAT indication: Endovascular infection	2.57	2.49
Dementia	2.55	1.14
OPAT indication: ENT infection	2.54	2.44
OPAT indication: Urinary tract infection	1.99	1.81
OPAT indication: Spinal infection	1.85	1.81
Peptic ulcer disease	1.82	1.07
Age	1.57	1.55
OPAT indication: Skin and soft tissue infection	1.56	1.55
Bed Days Saved (Duration of OPAT)	1.47	1.44
Type of vascular access	1.38	1.22
Male sex	1.23	1.22
**Average**	13.98	1.73
**Range**	1.23 - 239.81	1.03 - 5.47

Note. The variance inflation factor (VIF) quantifies the extent to which the variance of a regression coefficient for an explanatory variable is increased due to its correlation with other explanatory variables, compared with the variance expected in the absence of linear associations among predictors. As a rule of thumb, a VIF >5 suggests potential concern, while a VIF >10 indicates more serious multicollinearity that may adversely affect model estimation.

CCI, Charlson Comorbidity Index; ENT, ear, nose, and throat; IV, intravenous; OPAT, outpatient parenteral antimicrobial therapy; VIF, variance inflation factor

Supplementary Table S3. Intravenous antimicrobial agents administered during OPAT

IV Antimcrobial agent[a]	Total (n = 1124)	S-OPAT (n = 294)	H-OPAT (n = 689)	C-OPAT (n = 141)
Antifungal	3 (0.3)	1 (0.3)	-	2 (1.4)
Anidulafungin	1 (0.1)	1 (0.3)	-	-
Caspofungin	1 (0.1)	-	-	1 (0.7)
Rezafungin	1 (0.1)	-	-	1 (0.7)
Carbapenem	188 (16.7)	60 (20.4)	106 (15.4)	22 (15.6)
Ertapenem	162 (14.4)	45 (15.3)	96 (13.9)	21 (14.9)
Meropenem	26 (2.3)	15 (5.1)	10 (1.5)	1 (0.7)
Cephalosporin	176 (15.7)	54 (18.4)	100 (14.5)	22 (15.6)
Ceftazidime	16 (1.4)	6 (2.0)	8 (1.2)	2 (1.4)
Ceftriaxone	158 (14.1)	47 (16.0)	91 (13.2)	20 (14.2)
Cefuroxime	2 (0.2)	1 (0.3)	1 (0.1)	-
Glycopeptide	271 (24.1)	16 (5.4)	200 (29.0)	55 (39.0)
Dalbavancin	4 (0.4)	-	-	4 (2.8)
Teicoplanin	267 (23.8)	16 (5.4)	200 (29.0)	51 (36.2)
Penicillin	457 (40.7)	161 (54.8)	264 (38.3)	32 (22.7)
Flucloxacillin	139 (12.4)	55 (18.7)	70 (10.2)	14 (9.9)
Piperacillin/Tazobactam	318 (28.3)	106 (36.1)	194 (28.2)	18 (12.8)
Others	29 (2.6)	2 (0.7)	19 (2.8)	8 (5.7)
Daptomycin	28 (2.5)	2 (0.7)	19 (2.8)	7 (5.0)
Tigecycline	1 (0.1)	-	-	1 (0.7)

C-OPAT, clinic-based OPAT; H-OPAT, healthcare-administered OPAT at home; IV, intravenous; OPAT, outpatient parenteral antimicrobial therapy; S-OPAT, self/carer-administered OPAT.

[a] Some patients received more than one antimicrobial agent; therefore, the total number of antimicrobial agents exceeds the number of patient episodes. Percentages are calculated using the total number of antimicrobial agents as the denominator.

Supplementary Table S4. Description of clinical outcomes

Patient outcome	S-OPAT (n = 286)	H-OPAT (n = 646)	C-OPAT (n = 132)	Total (n = 1064)
Cure	14 (4.9)	32 (5.0)	3 (2.3)	49 (4.6)
Improved	246 (86.0)	502 (77.7)	115 (87.1)	863 (81.1)
Failure	26 (9.1)	112 (17.3)	14 (10.6)	152 (14.3)
Non-response of infection[a]	-	4 (0.6)	-	4 (0.4)
Death	-	1 (0.2)	-	1 (0.1)
Readmission	26 (9.1)	107 (16.6)	14 (10.6)	147 (13.8)
Adverse drug infection	2 (7.7)[b]	11 (10.3)[b]	1 (7.1)[b]	14 (9.5)[b]
New infection[c]	2 (7.7)[b]	17 (15.9)[b]	-	19 (12.9)[b]
Non-OPAT related	6 (23.1)[b]	34 (31.8 [b]	2 (14.3)[b]	42 (28.6)[b]
Surgical intervention	1 (3.8)[b]	5 (4.7)[b]	1 (7.1)[b]	7 (4.8)[b]
Vascular access related complications	5 (19.2)[b]	3 (2.8)[b]	1 (7.1)[b]	9 (6.1)[b]
Worsening of infection/no improvement	10 (38.5)[b]	37 (34.6)[b]	9 (64.3)[b]	56 (38.1)[b]

Data are presented as n (%).

C-OPAT, clinic-based OPAT; H-OPAT, healthcare-administered OPAT at home; OPAT, outpatient parenteral antimicrobial therapy; S-OPAT, self/carer-administered OPAT.

[a] Not requiring hospital readmission.

[b] As a percentage of number of readmissions.

[c] New infection included bacteria pneumonia, COVID-19 infection, and urinary tract infection.

Supplementary Table S5. Frequency of adverse events

**Type of adverse event**[a]	**S-OPAT**	**H-OPAT**	**C-OPAT**	**Total**
Drug-related adverse event	15	58	11	84
Antibiotic-induced diarrhoea[b]	3 (20.0)[c]	7 (12.1)[c]	1 (9.1)[c]	11 (13.1)[c]
Blood dyscrasia	1 (6.7)[c]	7 (12.1)[c]	1 (9.1)[c]	9 (10.7)[c]
Clostridiodes difficile infection	-	1 (1.7)[c]	-	1 (1.2)[c]
Drug Rash	2 (13.3)[c]	24 (41.4)[c]	2 (18.2)[c]	28 (33.3)[c]
Hepatic dysfunction	3 (20.0)[c]	1 (1.7)[c]	3 (27.3)[c]	7 (8.3)[c]
Other gastrointestinal disturbances[d]	1 (6.7)[c]	7 (12.1)[c]	1 (9.1)[c]	9 (10.7)[c]
Renal dysfunction	2 (13.3)[c]	6 (10.3)[c]	1 (9.1)[c]	9 (10.7)[c]
Other adverse drug events	3 (20.0)[c]	5 (8.6)[c]	2 (18.2)[c]	10 (11.9)[c]
Vascular access-related adverse event	33	54	6	93
Allergy to dressing	1 (3.0)[e]	-	1 (16.7)[e]	2 (2.2)[e]
Damaged line	4 (12.1)[e]	2 (3.7)[e]	1 (16.7)[e]	7 (7.5)[e]
Line infection	3 (9.1)[e]	-	-	3 (3.2)[e]
Line migration	6 (18.2)[e]	16 (29.6)[e]	-	22 (23.7)[e]
Line occlusion	15 (45.5)[e]	28 (51.9)[e]	2 (33.3)[e]	45 (48.4)[e]
Deep vein thrombus	2 (6.1)[e]	3 (5.6)[e]	1 (16.7)[e]	6 (6.5)[e]
Superficial thrombophlebitis	1 (3.0)[e]	3 (5.6)[e]	-	4 (4.3)[e]
Other vascular access complications	1 (3.0)[e]	2 (3.7)[e]	1(16.7)[e]	4 (4.3)[e]

Data are presented as n (%).

C-OPAT, clinic-based OPAT; H-OPAT, healthcare-administered OPAT at home; OPAT, outpatient parenteral antimicrobial therapy; S-OPAT, self/carer-administered OPAT.

[a] Some patient-episodes had more than one adverse event.

[b] Antibiotic-induced diarrhoea excluded *Clostridiodes difficile*-associated diarrhoea.

[c] As a percentage of number of drug-related adverse events.

[d] Other gastrointestinal disturbances included nausea, vomiting, dyspepsia, abdominal discomfort, and bloating.

[e] As a percentage of number of vascular access related adverse events.

Supplementary Table S6. Relative risk regression analysis of the association between OPAT delivery models and treatment failure in 1064 OPAT episodes from 846 distinct patients

**Treatment failure**	**Adj. RR (95% CI)**	**Pairwise *P* value**	**Omnibus *P* value**
OPAT delivery model			
Self/carer-administered	0.94 (0.54 - 1.63)	0.825	0.217
Healthcare-administered at home	1.31 (0.81 - 2.12)	0.266	
Clinic-based	1.00		
Sex			
Female	1.00		
Male	0.74 (0.55 - 0.99)	0.045	
Age (per year increase)	1.01 (0.99 - 1.02)	0.350	
Charlson comorbidity index group			
0-1	1.00		< 0.001
2	2.15 (1.32 - 3.49)	0.002	
3-4	1.82 (1.14 - 2.91)	0.013	
5+	3.86 (2.62 - 5.69)	< 0.001	
Indication for OPAT			
Bone and joint infection	1.00		0.010
Respiratory infection	0.45 (0.25 - 0.82)	0.009	
Endovascular infection	0.90 (0.56 - 1.44)	0.650	
ENT infection	0.58 (0.32 - 1.07)	0.079	
Urinary tract infection	0.71 (0.38 - 1.31)	0.272	
Spinal infection	1.50 (0.89 - 2.51)	0.125	
Skin and soft tissue infection	0.21 (0.03 - 1.41)	0.107	
Other indication	0.48 (0.18 - 1.31)	0.154	
Concomitant IV antimicrobial therapy			
No	1.00		
Yes	0.59 (0.27 - 1.26)	0.170	
Type of vascular access			
Peripheral access	1.00		
Central line	1.19 (0.84 - 1.69)	0.328	
Intercept	0.06 (0.02 - 0.16)	< 0.001	

The adjusted risk ratio was derived from a robust Poisson regression model using generalised estimating equations to account for correlation between patients with multiple OPAT episodes. Omnibus *P* values for categorical variables test the null hypothesis of equal risk across all levels of the factor.

Adj. RR, adjusted risk ratio; CI, confidence interval; ENT, ear, nose, and throat; IV, intravenous; OPAT, outpatient parenteral antimicrobial therapy.

Supplementary Table S7. Relative risk regression analysis of the association between OPAT delivery models and risk of any OPAT-related adverse event in 1064 OPAT episodes from 846 distinct patients

**Any OPAT-related adverse event based**	**Adj. RR (95% CI)**	**Pairwise *P* value**	**Omnibus *P* value**
OPAT delivery model			
Self/carer-administered	1.38 (0.79 - 2.42)	0.256	0.459
Healthcare-administered at home	1.38 (0.81 - 2.36)	0.230	
Clinic-based	1.00		
Sex			
Female	1.00		
Male	0.80 (0.60 - 1.07)	0.134	
Age (per year increase)	1.00 (0.99 - 1.01)	0.851	
Charlson comorbidity index group			
0-1	1.00		0.901
2	0.91 (0.59 - 1.39)	0.657	
3-4	0.88 (0.60 - 1.29)	0.518	
5+	0.89 (0.59 - 1.35)	0.590	
Indication for OPAT			
Bone and joint infection	1.00		< 0.001
Respiratory infection	0.43 (0.25 - 0.73)	0.002	
Endovascular infection	0.90 (0.55 - 1.45)	0.653	
ENT infection	2.22 (1.38 - 3.56)	< 0.001	
Urinary tract infection	0.50 (0.21 - 1.22)	0.129	
Spinal infection	1.44 (0.87 - 2.39)	0.153	
Skin and soft tissue infection	0.71 (0.31 - 1.61)	0.412	
Other indication	0.79 (0.43 - 1.47)	0.465	
Concomitant IV antimicrobial therapy			
No	1.00		
Yes	1.45 (0.89 - 2.34)	0.134	
Type of vascular access			
Peripheral access	1.00		
Central line	0.80 (0.55 - 1.17)	0.257	
Intercept	0.17 (0.08 - 0.35)	< 0.001	

The adjusted risk ratio was derived from a robust Poisson regression model using generalised estimating equations to account for correlation between patients with multiple OPAT episodes. Omnibus *P* values for categorical variables test the null hypothesis of equal risk across all levels of the factor.

Adj. RR, adjusted risk ratio; CI, confidence interval; ENT, ear, nose, and throat; IV, intravenous; OPAT, outpatient parenteral antimicrobial therapy.

Supplementary Table S8. Relative risk regression analysis of the association between OPAT delivery models and risk of medication-related adverse events in 1064 OPAT episodes from 846 distinct patients

Medication-related adverse event	Adj. RR (95% CI)	Pairwise *P* value	Omnibus *P* value
OPAT delivery model			
Self/carer-administered	0.63 (0.29 - 1.36)	0.237	0.337
Healthcare-administered at home	1.01 (0.53 - 1.93)	0.979	
Clinic-based	1.00		
Sex			
Female	1.00		
Male	0.84 (0.55 - 1.28)	0.418	
Age (per year increase)	1.00 (0.99 - 1.02)	0.816	
Charlson comorbidity index group			
0-1	1.00		0.643
2	1.21 (0.67 - 2.17)	0.528	
3-4	0.77 (0.43 - 1.39)	0.391	
5+	1.04 (0.58 - 1.85)	0.905	
Indication for OPAT			
Bone and joint infection	1.00		0.014
Respiratory infection	0.53 (0.25 - 1.11)	0.094	
Endovascular infection	1.31 (0.69 - 2.45)	0.408	
ENT infection	2.76 (1.33 - 5.75)	0.007	
Urinary tract infection	0.48 (0.11 - 2.07)	0.325	
Spinal infection	1.29 (0.55 - 3.01)	0.561	
Skin and soft tissue infection	0.67 (0.17 - 2.71)	0.574	
Other indication	0.58 (0.18 - 1.85)	0.355	
Concomitant IV antimicrobial therapy			
No	1.00		
Yes	1.95 (1.02 - 3.74)	0.044	
Type of vascular access			
Peripheral access	1.00		
Central line	1.10 (0.65 - 1.85)	0.718	
Intercept	0.08 (0.03 - 0.22)	< 0.001	

The adjusted risk ratio was derived from a robust Poisson regression model using generalised estimating equations to account for correlation between patients with multiple OPAT episodes. Omnibus *P* values for categorical variables test the null hypothesis of equal risk across all levels of the factor.

Adj. RR, adjusted risk ratio; CI, confidence interval; ENT, ear, nose, and throat; IV, intravenous; OPAT, outpatient parenteral antimicrobial therapy.

Supplementary Table S9. Relative risk regression analysis of the association between OPAT delivery models and risk of vascular access-related adverse events in 1064 OPAT episodes from 846 distinct patients

**Vascular access-related adverse event**	**Adj. RR (95% CI)**	**Pairwise *P* value**	**Omnibus *P* value**
OPAT delivery model			
Self/carer-administered	2.99 (1.19 - 7.52)	0.020	0.034
Healthcare-administered at home	2.01 (0.76 - 5.31)	0.157	
Clinic-based	1.00		
Sex			
Female	1.00		
Male	0.75 (0.49 - 1.15)	0.187	
Age (per year increase)	1.00 (0.98 - 1.01)	0.752	
Charlson comorbidity index group			
0-1	1.00		0.605
2	0.66 (0.30 - 1.47)	0.308	
3-4	1.00 (0.58 - 1.71)	> 0.99	
5+	0.73 (0.38 - 1.40)	0.344	
Indication for OPAT			
Bone and joint infection	1.00		0.003
Respiratory infection	0.36 (0.17 - 0.76)	0.007	
Endovascular infection	0.45 (0.18 - 1.13)	0.089	
ENT infection	1.94 (0.99 - 3.77)	0.053	
Urinary tract infection	0.45 (0.13 - 1.50)	0.192	
Spinal infection	1.46 (0.70 - 3.01)	0.312	
Skin and soft tissue infection	0.65 (0.20 - 2.12)	0.478	
Other indication	0.85 (0.39 - 1.86)	0.681	
Concomitant IV antimicrobial therapy			
No	1.00		
Yes	1.26 (0.56 - 2.85)	0.578	
Type of vascular access			
Peripheral access	1.00		
Central line	0.58 (0.33 - 1.01)	0.054	
Intercept	0.08 (0.03 - 0.24)	< 0.001	

The adjusted risk ratio was derived from a robust Poisson regression model using generalised estimating equations to account for correlation between patients with multiple OPAT episodes. Omnibus *P* values for categorical variables test the null hypothesis of equal risk across all levels of the factor.

Adj. RR, adjusted risk ratio; CI, confidence interval; ENT, ear, nose, and throat; IV, intravenous; OPAT, outpatient parenteral antimicrobial therapy.

Supplementary Table S10. Relative risk regression analysis of the association between OPAT delivery models and 30-day unplanned hospitalisation in 1064 OPAT episodes from 846 distinct patients

**30-day unplanned hospitalisation**	**Adj. RR (95% CI)**	**Pairwise *P* value**	**Omnibus *P* value**
OPAT delivery model			
Self/carer-administered	0.96 (0.64 - 1.45)	0.849	0.476
Healthcare-administered at home	1.14 (0.79 - 1.65)	0.489	
Clinic-based	1.00		
Sex			
Female	1.00		
Male	0.82 (0.64 - 1.04)	0.099	
Age (per year increase)	1.00 (0.99 - 1.01)	0.442	
Charlson comorbidity index group			
0-1	1.00		<0.001
2	1.72 (1.20 - 2.46)	0.003	
3-4	1.82 (1.31 - 2.53)	<0.001	
5+	2.47 (1.82 - 3.36)	<0.001	
Indication for OPAT			
Bone and joint infection	1.00		0.085
Respiratory infection	0.91 (0.64 - 1.29)	0.587	
Endovascular infection	0.88 (0.58 - 1.32)	0.526	
ENT infection	0.53 (0.31 - 0.90)	0.019	
Urinary tract infection	0.84 (0.50 - 1.41)	0.505	
Spinal infection	1.30 (0.85 - 2.00)	0.231	
Skin and soft tissue infection	0.28 (0.07 - 1.06)	0.061	
Other indication	0.91 (0.55 - 1.50)	0.701	
Concomitant IV antimicrobial therapy			
No	1.00		
Yes	0.58 (0.31 - 1.07)	0.080	
Type of vascular access			

Peripheral access	1.00	
Central line	1.21 (0.91 - 1.60)	0.190
Intercept	0.13 (0.07 - 0.25)	<0.001

The adjusted risk ratio was derived from a robust Poisson regression model using generalised estimating equations to account for correlation between patients with multiple OPAT episodes. Omnibus *P* values for categorical variables test the null hypothesis of equal risk across all levels of the factor.

Adj. RR, adjusted risk ratio; CI, confidence interval; ENT, ear, nose, and throat; IV, intravenous; OPAT, outpatient parenteral antimicrobial therapy.

Supplementary Figure S1. Graphical assessment of the linearity of the relationship between clinical outcomes (treatment failure, OPAT-related adverse events, and 30-day unplanned hospitalisation) and continuous covariates (age and Charlson comorbidity index score).

For each outcome, three multivariable GEE-based robust Poisson regression models were fitted, incorporating different functional forms for each continuous covariate (log-linear, categorical based on quartile cut-offs, and flexible models using restricted cubic splines with four knots), while adjusting for other covariates (patient sex, indication for OPAT, concomitant IV antimicrobial therapy, and type of vascular access device).

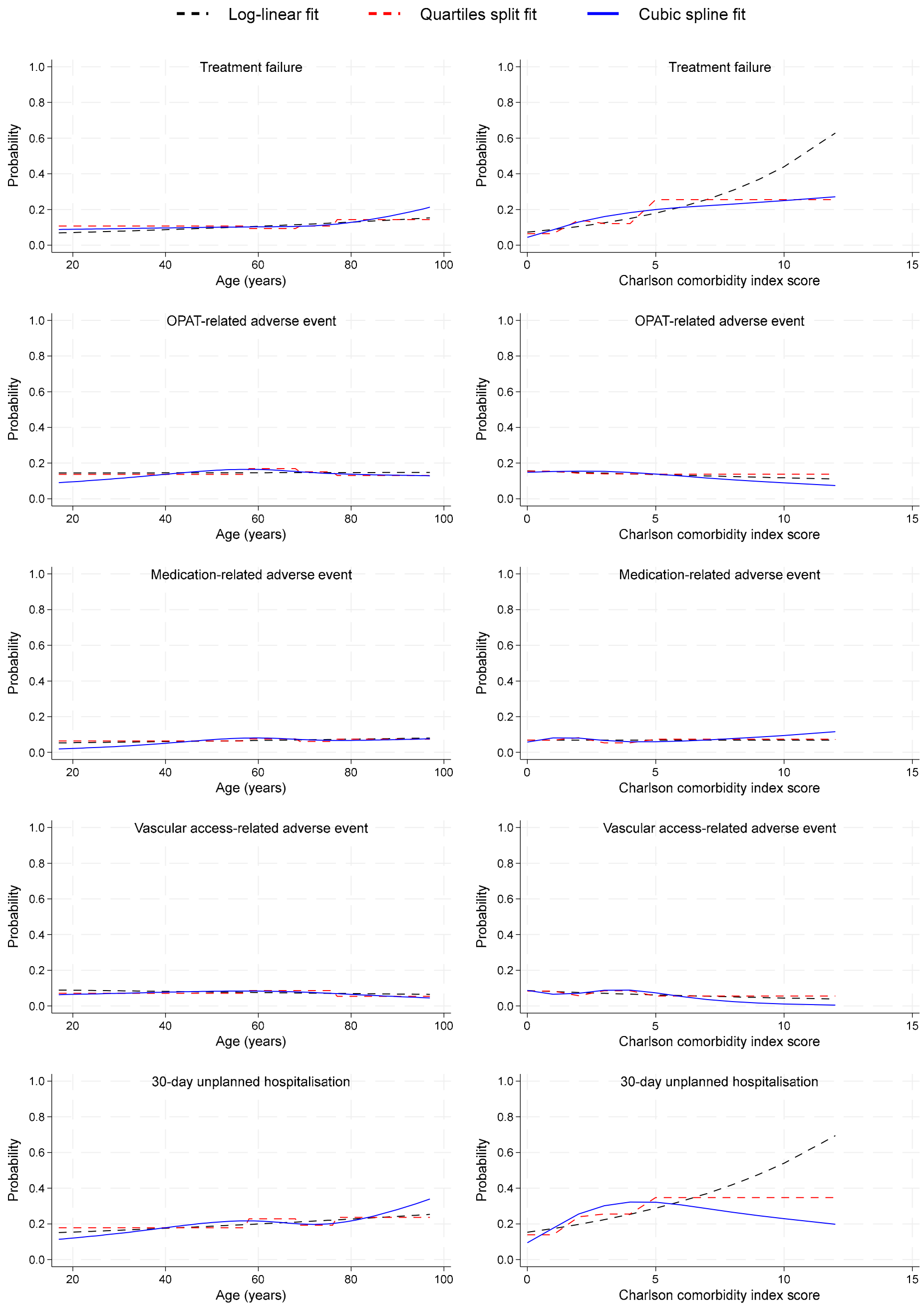
Log-linear fit
Quartiles split fit
Cubic spline fit
Treatment failure
Probability
Age (years)
Charlson comorbidity index score
OPAT-related adverse event
Medication-related adverse event
Vascular access-related adverse event
30-day unplanned hospitalisation

GEE, generalised estimating equations; IV, intravenous; OPAT, outpatient parenteral antimicrobial therapy

Supplementary Figure S2. Comparison of crude (unadjusted) clinical outcomes across OPAT delivery models

Outcome endpoint	Contrast	Crude RR (98.3% CI)	Pairwise adj. P	Omnibus P
**Treatment failure**	S- vs H-OPAT	0.53 (0.32, 0.87)	0.007	0.003
	S- vs C-OPAT	0.90 (0.44, 1.85)	>0.99	
	C- vs H-OPAT	0.59 (0.32, 1.08)	0.11	
**OPAT-related adverse event**	S- vs H-OPAT	0.96 (0.64, 1.43)	>0.99	0.45
	S- vs C-OPAT	1.32 (0.68, 2.57)	0.95	
	C- vs H-OPAT	0.73 (0.40, 1.33)	0.62	
**Medication-related adverse event**	S- vs H-OPAT	0.57 (0.27, 1.18)	0.20	0.18
	S- vs C-OPAT	0.63 (0.25, 1.63)	0.74	
	C- vs H-OPAT	0.90 (0.43, 1.90)	>0.99	
**Vascular access-related adverse event**	S- vs H-OPAT	1.48 (0.89, 2.45)	0.19	0.028
	S- vs C-OPAT	2.85 (0.99, 8.23)	0.054	
	C- vs H-OPAT	0.52 (0.18, 1.46)	0.39	
**30-day unplanned hospitalisation**	S- vs H-OPAT	0.72 (0.50, 1.03)	0.088	0.041
	S- vs C-OPAT	0.98 (0.58, 1.67)	>0.99	
	C- vs H-OPAT	0.73 (0.46, 1.15)	0.30	

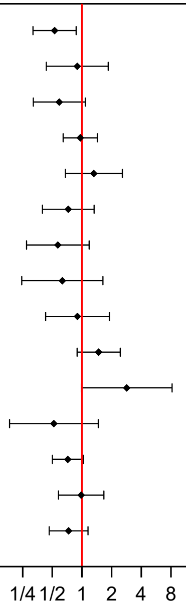

Adj. P, adjusted *P*-value; C-OPAT, clinic-based OPAT; CI, confidence interval; H-OPAT, healthcare-administered OPAT at home; OPAT, outpatient parenteral antimicrobial therapy; RR, risk ratio; S-OPAT, self/carer-administered OPAT.
